# The pulvinar regulates plasticity in human visual cortex

**DOI:** 10.1126/sciadv.adw9988

**Published:** 2025-11-28

**Authors:** Miriam Acquafredda, Jan W. Kurzawski, Laura Biagi, Michela Tosetti, Maria Concetta Morrone, Paola Binda

**Affiliations:** ^1^ Department of Translational Research and New Technologies in Medicine and Surgery, University of Pisa, Pisa, Italy.; ^2^ Faculty of Psychology and Neuroscience, Maastricht University, Maastricht, Netherlands.; ^3^ IRCCS Stella Maris, Calambrone, Pisa, Italy.; ^4^ IMAGO7 Center, Pisa, Italy.

## Abstract

In normally sighted human adults, 2 hours of monocular deprivation is sufficient to transiently alter ocular dominance. Here, we show that this is associated with a reduction of functional connectivity between the pulvinar and primary visual cortex (V1), selective for the pulvinar-to-V1 directionality. Across participants, the strength of the pulvinar-to-V1 connectivity was negatively correlated with the ocular dominance shift, implying less plasticity in participants with stronger influence of the pulvinar over V1. Our results support a revised model of adult V1 plasticity, where short-term reorganization is gated by modulatory signals relayed by the pulvinar.

## Teaser

Short-term monocular deprivation triggers reorganization in the visual processing network measured with 7-T fMRI in human adults.

## INTRODUCTION

The potential for long-term plastic change in the sensory brain declines with age, probably through active stabilization of the sensory circuitry (*1*). Nevertheless, adult sensory systems retain the potential for short-term plasticity (*2*, *3*): Blocking vision in one eye for a few hours in human adults induces a transient boost of the deprived eye (*4*, *5*). This response modulation was observed using functional magnetic resonance imaging (fMRI) in primary visual cortex V1 (*6*) but not in the lateral geniculate nucleus (LGN) (*7*), indicating that the modulation of cortical activity was not inherited from earlier stages. Visual responses were also modulated in an adjacent thalamic nucleus, the pulvinar (*7*); whether this pulvinar modulation was a product of the V1 signal change or involved in its generation remained an open question. The homeostatic response reflects a gain change of the deprived eye representation, which could serve to counterbalance the reduced stimulation (*8*). However, there is recent evidence that the same boost can be achieved without blocking stimulation of either eye, merely manipulating attention and multimodal context (*9*–*12*). This suggests an innovative interpretation of adult plasticity, where top-down multimodal signals play a key role.

One main pathway through which multimodal signals reach V1 is via the pulvinar, which modulates activity through recurrent cortico-thalamo-cortical loops (*13*, *14*). These loops connect distant sensory, motor, and cognitive areas and may underly predictive coding (*15*–*17*): By collecting multimodal signals, the pulvinar may carry predictions about the upcoming sensory events to low-level sensory areas, including V1 (*18*, *19*). In line with this model, there is evidence that reducing the pulvinar influence over V1 permits the updating of perceptual representations, i.e., perceptual learning (*20*).

On the basis of this evidence, we hypothesize that the pulvinar exerts a stabilizing influence over the primary sensory cortex and that reducing this influence may open an opportunity for plasticity—even for a very stable property such as ocular dominance. To test this idea, we used high-resolution 7-T fMRI and asked whether short-term monocular deprivation affects the pulvinar-V1 connectivity.

## RESULTS

### Short-term monocular deprivation alters pulvino-cortical functional connectivity

We first confirmed the deprivation effect in 22 normally sighted adults. After the 2-hour monocular deprivation, ocular dominance, measured with a short binocular rivalry test immediately before MRI scanning, shifted in favor of the deprived eye by 10% on average (from 55 to 65% dominance).

**Fig. 1.**
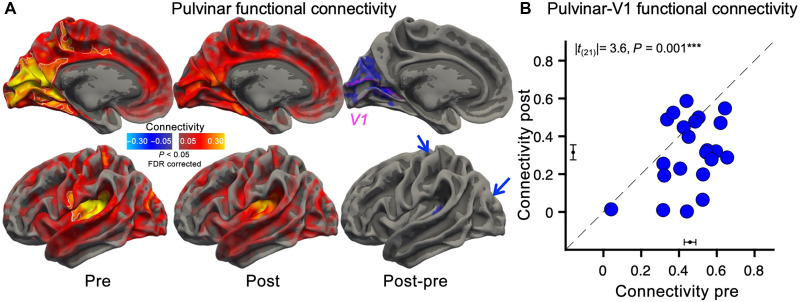
Functional connectivity of the pulvinar, measured with resting-state 7-T fMRI. (**A**) Functional connectivity of the pulvinar, estimated from resting-state fMRI (TR = 3 s; full brain coverage) and mapped over the cortical surface (medial and lateral view; maps are averaged across hemispheres and projected on a symmetrical template of the cortical surface). The leftmost and central maps show the connectivity before and after 2 hours of monocular deprivation; the rightmost maps show the connectivity change, showing significant reductions in occipital visual areas (mainly mesial and one postero-lateral, not visible and signaled by the posterior blue arrow); there was also a connectivity reduction in two small areas of the superior temporal gyrus (visible in the lateral view) and the central sulcus (signaled by the anterior blue arrow). Connectivity was estimated as the Pearson’s *r* coefficient between the fMRI time series in the pulvinar and the time series of each cortical voxel, computed after nuisance regression and concatenation across *N* = 22 participants. Maps are thresholded at |*r*| > 0.05, *P* < 0.05 false discovery rate (FDR) corrected, and cluster corrected (50 vertices). The white outlines in the predeprivation map identify the clusters of regions where connectivity is highly reliable (|*r*| > 0.20 and 500 vertices); the pink outline in the map of connectivity differences shows the V1 ROI. (**B**) Functional connectivity between the pulvinar and V1 [atlas-based definition; see pink outline in (A)] before and after deprivation for the individual participants. Pearson’s *r* values were Fisher transformed to allow for statistical comparisons; the black error bars near the axes show the mean ± SEM connectivity: 0.46 ± 0.03 before deprivation reduces to 0.32 ± 0.04 after deprivation. The text inset reports the results of the paired *t* test, comparing functional connectivity before and after deprivation (Cohen’s *d* = 0.80).

Next we measured the resting-state (eyes closed) functional connectivity between the pulvinar [atlas-based region-of-interest (ROI) definition (*21*)] and the cortex; this was strong in all sensory regions, including occipital visual areas, temporal auditory areas, and central somatosensory areas (Fig. 1A, left) in four highly significant clusters (continuous white lines), consistent with the multimodality of the pulvinar (*22*, *23*). After monocular deprivation (Fig. 1A, middle), there was a marked reduction of pulvinar connectivity with the occipital cortex, including the primary visual cortex V1 and surrounding early visual areas (Fig. 1A, right, displaying the post-pre deprivation difference) and fewer and sparser reductions in the temporal and parietal cortex.

**Fig. 2.**
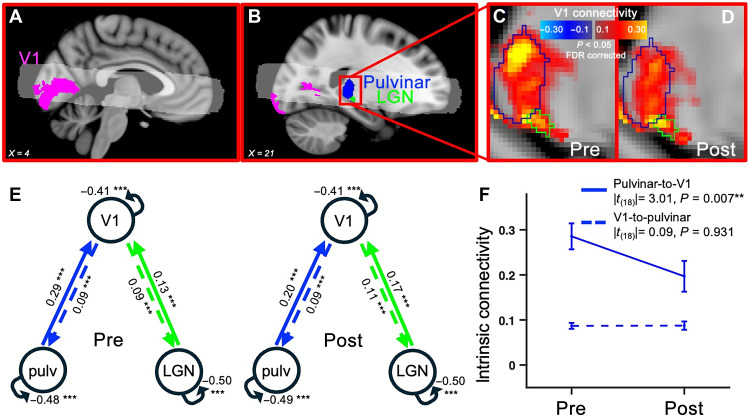
Thalamus-V1 functional connectivity and effective connectivity measured with DMC of the resting-state 7-T fMRI time series. (**A** and **B**) High-temporal resolution fMRI (TR = 1 s, partial volume: white shading shows coverage across 50% of our acquisitions) used for measuring connectivity between V1 [defined as the intersection between the coverage and the atlas-based V1 ROI (*24*); in pink] and the pulvinar and LGN [atlas-based definitions (*21*, *25*); blue and green masks], visualized in two different sagittal slices [*x*- Montreal Neurological Institute (MNI) coordinates in text insets]. (**C** and **D**) Functional connectivity between V1 and the thalamus (TR = 1 s), before and after monocular deprivation. Connectivity was estimated as the Pearson’s *r* coefficient between the fMRI time series in V1 and the time series of each voxel in the thalamus, computed after nuisance regression and concatenation across *N* = 19 participants; maps are thresholded at |*r*| > 0.05 and *P* < 0.05 FDR corrected. See fig. S5 (A and B) for a coronal view of the V1 connectivity profile within the pulvinar ROI. (**E**) Average effective connectivity in the network including bidirectional connections between V1 and the two thalamic regions: pulvinar and LGN. Numbers show intrinsic connectivity [A matrix in regression DCM (rDCM)] and associated statistical significance [after FDR correction: *P < 0.05, **P < 0.01, ***P < 0.001, and not significant (n.s.) for *P* > = 0.05], independently estimated before and after monocular deprivation. pulv, pulvinar. (**F**) Change of effective connectivity between the pulvinar and V1, shown as mean [same numbers as in (E)] and SEM across participants. Text insets report the post hoc *t* tests comparing effective connectivity values before and after deprivation for the two directionalities (after FDR correction: *P < 0.05 and **P< 0.01). Figure S4A shows results for an alternative DCM, assuming reciprocal connectivity between all areas (including LGN and pulvinar) and characterized by a worse fit to the data.

This connectivity reduction after deprivation was confirmed by calculating functional connectivity for the individual participants (Fig. 1B), from the average time series of two a-priori selected ROIs: pulvinar (illustrated in Fig. 2B) (*21*) and V1 (illustrated by the pink outline in Fig. 1A and the pink region in Fig. 2A) (*24*). The connectivity change was not accompanied by changes in blood oxygen level–dependent (BOLD) modulations, as the overall power of the Fourier spectrum of the signals in each ROI remained comparable before and after deprivation in all ROIs [V1: |*t*_(21)_| = 0.3, *P* = 0.803, and Cohen’s *d* = 0.05; pulvinar: |*t*_(21)_| = 1.4, *P* = 0.173, and Cohen’s *d* = 0.30]. The change in pulvinar connectivity extended beyond V1 to several other visual areas (fig. S3A) and to few areas of the superior temporal gyrus and central sulcus (fig. S3A). In contrast with the marked reduction of pulvinar connectivity, no change was observed for LGN connectivity (fig. S1, A and B) (*25*) nor for connectivity between V1 and the other occipital areas (fig. S2). There was, however, a sparse reduction of V1 connectivity with high-level areas, distributed across the superior temporal gyrus, the intraparietal sulcus, the premotor cortex, and the cingulate gyrus (fig. S2).

### The functional connectivity reduction is specific for the pulvinar-to-V1 directionality

To estimate the directionality of the pulvinar-V1 connectivity change (feedforward versus feedback), we acquired a second resting-state fMRI dataset, with higher temporal resolution (TR = 1 s, covering V1 and the thalamus; Fig. 2, A and B). The V1 functional connectivity with the thalamus (Fig. 2C) shows strong connectivity both in the atlas-based definition of LGN (*25*) and in the atlas-based definition of the pulvinar (*21*). Comparison of the V1 connectivity map before and after deprivation (Fig. 2, C and D) suggests a selective reduction in the pulvinar, but not LGN. We applied dynamic causal modeling (DCM) (*26*) to estimate bidirectional effective connectivity among the three regions: V1, LGN, and pulvinar. We tested two models, one fully connected (fig. S4A) and a more anatomically plausible model with no direct connections between LGN and pulvinar, as shown in Fig. 2E. The latter showed a better fit to the data than the fully connected model [negative free energy |*t*_(18)_| = 88.7, *P* < 0.001, and Cohen’s *d* = 20.3]. Effective connectivity estimates from this model on the individual participants’ data (Fig. 2, E and F) show that monocular deprivation selectively reduced the pulvinar-to-V1, leaving the LGN-to-V1 connectivity and the connectivity from V1 to either of the thalamic regions unaffected. A two-by-two analysis of variance (ANOVA) with factors time (pre- versus postdeprivation) and directionality (thalamus-to-V1 versus V1-to-thalamus) showed a significant interaction for the effective connectivity between the pulvinar and V1 [*F*_(1,18)_ = 8.30, *P* = 0.010, and η^2^_partial_ = 0.32], not between LGN and V1 [*F*_(1,18)_ = 0.2, *P* = 0.638, and η^2^_partial_ = 0.01]; post hoc *t* tests comparing effective connectivity values before and after deprivation for the two directionalities showed significant reduction of pulvinar-to-V1 connectivity [|*t*_(18)_| = 3.01, *P* = 0.007, and Cohen’s *d* = 0.69], no change of V1-to-pulvinar connectivity [|*t*_(18)_| = 0.09, *P* = 0.931, and Cohen’s *d* = 0.02], and no change for LGN-to-V1 [|*t*_(18)_| = 0.75, *P* = 0.464, and Cohen’s *d* = 0.17] or V1-to-LGN connectivity [|*t*_(*1*8)_| = 0.51, *P* = 0.617, and Cohen’s *d* = 0.12]. Similar results hold for the fully connected model (fig. S4A), although this model assigns significant connectivity values to the LGN-pulvinar connection before deprivation, which is not supported by anatomical evidence [e.g., (*27*)]. The DCM-based conclusions are also supported by a more direct cross-correlation analysis of the BOLD time series (shown in fig. S4B).

**Fig. 3.**
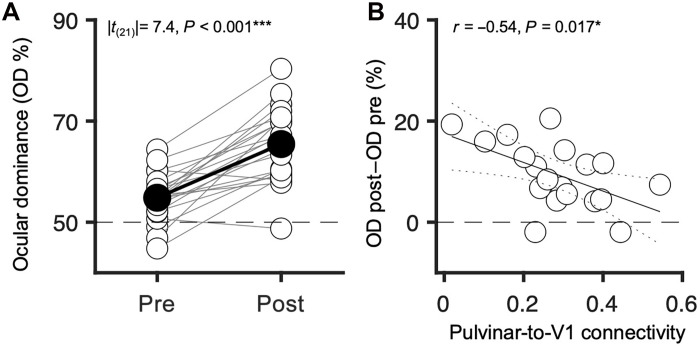
Ocular dominance plasticity correlates with pulvinar-to-V1 connectivity. (**A**) Ocular dominance measured with binocular rivalry before and after the 2-hour monocular deprivation, shifting in favor of the deprived eye. Each empty symbol is one participant; pre- and postdeprivation values are connected by thin lines. Filled symbols and the thick line show the average across participants, and the paired comparison across time points is reported in the text inset. (**B**) In the subset of 19 participants included in the dynamic causal modeling (DCM) analysis, the ocular dominance shift was negatively correlated with the pulvinar-to-V1 connectivity estimated with DCM from predeprivation acquisitions. The text inset gives the Pearson’s correlation coefficient and associated *P* value. Continuous and dotted lines show the best-fitting linear function and the 95% confidence bands.

The DCM estimate of pulvinar-to-V1 connectivity was related to ocular dominance measured behaviorally (shown in Fig. 3A). The variability across participants in the strength of the pulvinar-to-V1 connectivity before deprivation reliably predicted the change of ocular dominance with monocular deprivation (Fig. 3B). The correlation is negative: participants with stronger pulvinar influence over V1 showed less ocular dominance plasticity. We did not observe a reliable correlation between connectivity changes and the change of ocular dominance (*r* = −0.05 and *P* = 0.834), probably reflecting the increase in error associated with the difference between the pre- and postdeprivation noisy estimates of connectivity.

The V1 connectivity map in the pulvinar and its change with deprivation showed at least two distinguishable foci (fig. S5, A and B). To gain further insight into possible subdivisions of the pulvinar region (Fig. 2, C and D), we recorded visual BOLD responses in two conditions differing in voluntary attention allocation (fig. S5C). We used stimuli that reliably mapped thalamic activations (*7*); these were presented either in passive viewing or while participants allocated attention to the stimulus area to detect small peripheral color changes. As expected, passive viewing elicited a cluster of activity within the ventral portion of the pulvinar, and attending to the stimuli elicited additional activity in a more dorsal portion. We used these activity maps to define a ventral visual pulvinar (cyan outline in fig. S5, A to C) and a relatively dorsal pulvinar modulated by attention allocation (magenta outline). The union of these two sub-ROIs covered about half the anatomically defined pulvinar (*21*), and the connectivity of this region with V1 was reliably stronger than in the remaining half of the pulvinar [|*t*_(18)_| = 5.7, *P* < 0.001, and Cohen’s *d* = 1.30]. By applying our DCM-based approach to each subregion, we observed that the reduction of pulvinar-to-V1 connectivity was present only for the relatively dorsal region (fig. S5, D and E), supporting the implication of a high-level mechanism.

### Short-term monocular deprivation does not affect thalamo-cortical structural connectivity

**Fig. 4.**
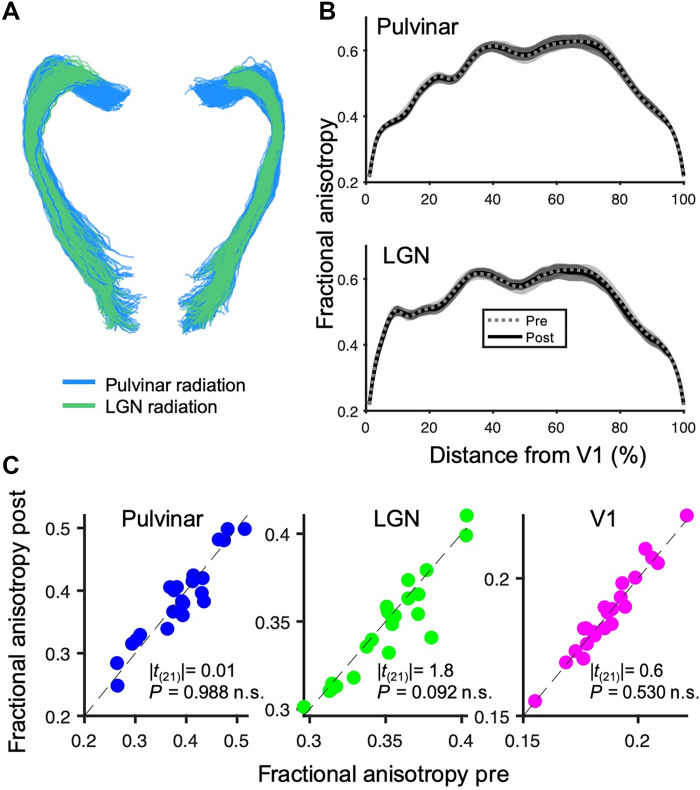
Structural connectivity between the thalamus and V1, measured with DWI. (**A**) Fiber tracking (visualized in DSI Studio) for a representative participant, mapping the structural connectivity between V1 and two thalamic regions: LGN and pulvinar. (**B**) Fractional anisotropy values as a function of nodes along the tracts (averaged across hemispheres), measured before (dashed gray lines) and after monocular deprivation (continuous black lines); shaded areas show 1 SEM across participants. No difference was detected before versus after monocular deprivation (paired *t* tests; all *P* > 0.05 FDR corrected). (**C**) Fractional anisotropy before versus after monocular deprivation within each region for the individual participants. Text insets report the results of paired *t* tests comparing fractional anisotropy before and after deprivation (Cohen’s *d* = 0.00, 0.38, and 0.14 for pulvinar, LGN, and V1).

We also assessed whether these functional connectivity changes are associated with changes in structural connectivity. We acquired diffusion-weighted MR images (DWI) during the same sessions as the resting-state scans. Figure 4 shows that thalamus-V1 structural connectivity was unaffected by monocular deprivation.

## DISCUSSION

Our results show that 2 hours of monocular deprivation reshapes the reciprocal interactions between visual brain regions, decreasing the influence of the pulvinar on resting-state V1 activity. In contrast, monocular deprivation did not reduce the LGN bottom-up sensory drive to V1.

Functional connectivity between the pulvinar and the cortex was reduced in both V1 and other visual areas, mainly in the dorsal mesial occipital cortex, with sparser connectivity changes in the auditory and somatosensory cortex. In V1, where effective connectivity could be estimated at high temporal resolution, the reduction was selective for the pulvinar-to-cortex connectivity, while connectivity in the cortex-to-pulvinar direction was stable. A similar trend was observed for the only other cortical area covered by our high-temporal resolution acquisitions that showed significantly reduced functional connectivity with the pulvinar, corresponding with the auditory belt area. We also analyzed the topography of functional connectivity between V1 and individual voxels within the pulvinar and found that connectivity changes were primarily localized within a dorsal subregion of the pulvinar. This region was defined by its modulation with attention, implicating the fronto-parietal network (*13*, *28*) and top-down signals in the monocular deprivation effect. The topography of the effects, both across the cortex and within the pulvinar, is consistent with modulation by top-down contextual signals accompanying plasticity in early visual cortex.

The short-term effects of monocular deprivation in adults that we study here are very different from the long-term effects observed during development, when permanent suppression of the deprived-eye representation is accompanied by reduced bottom-up drive from LGN (*1*). The counterintuitive and opposite effect elicited by short-term deprivation (deprived-eye enhancement) is often interpreted as a homeostatic response to the reduced sensory input in the deprived eye (*8*, *29*). Our results add to previous evidence (*7*) indicating that the gain change is not accompanied by a modulation of the bottom-up input from LGN; they suggest that it is associated with decreased top-down influences conveyed through the pulvinar—primarily a dorsal subregion of the pulvinar. We find that the strength of the pulvinar-to-V1 connectivity predicts the participants’ behavioral susceptibility to plastic changes, demonstrating a functional role of this pulvinar-to-V1 communication. These observations are consistent with the hypothesis that the pulvinar modulates the neuronal gain in V1, gating its homeostatic plasticity response.

Given that pulvinar-to-V1 projections can exert an inhibitory effect (*30*), the reduced pulvinar connectivity is consistent with the evidence that monocular deprivation reduces resting γ-aminobutyric acid (GABA) in V1 (*31*) and that monocular deprivation enhances visual evoked and fMRI responses in the visual cortex (*6*, *32*), suggesting that plasticity may be associated with increased cortical excitability. There is also evidence for increased excitability in the visual cortex following short-term binocular deprivation [blindfolding (*33*, *34*)], but whether this other form of short-term plasticity is accompanied by decreased pulvinar-to-V1 connectivity is currently unknown. Thus, available evidence is suggestive of a relation between short-term deprivation, shifted excitation/inhibition balance, and pulvinar connectivity, but demonstrating the link among these phenomena remains a goal for future research.

We hypothesize that a downweighting of pulvinar inputs to V1 may be a common trait of diverse adult V1 plasticity phenomena, including monocular deprivation and perceptual learning, as both are associated with reduced pulvinar-V1 connectivity and decreased cortical GABA (*20*, *31*). While perceptual learning is accompanied by structural plasticity (*20*) [such as motor learning (*35*)], we found that monocular deprivation does not affect structural connectivity (assessed through DWI). This suggests that structural plasticity may require a longer period of altered experience than the mere 2 hours of our deprivation.

Although the modulation of pulvinar functional connectivity was mainly localized within the visual cortex, sparser but significant connectivity reductions were also observed in auditory and somatosensory areas. This is in line with evidence that monocular deprivation affects multisensory processing (*36*, *37*). This modulation could be part of a recalibration response, which may be required to preserve the alignment across modalities whenever processing in one sensory modality is altered. Our results do not offer insights into the mechanisms implementing these connectivity changes. Strong functional connectivity can either be supported by direct communication between two regions, or it can be mediated by a third region that is strongly connected with both. This implies that auditory and somatosensory areas may show a deprivation effect due to their common input from cortical, thalamic, or brainstem circuitry, leaving open the interpretation of this result.

On the basis of the knowledge that the pulvinar is crucial for conveying modulatory inputs to early sensory areas (*15*, *16*, *18*), we suggest that a primary effect of monocular deprivation is to reduce the impact of top-down signals on early visual processing. This is in line with previous evidence of impaired multisensory integration (*36*) and reduced feedback signaling (alpha rhythms) (*32*, *37*) after monocular deprivation. The reduction of top-down influences could result from the sensory conflict generated by deprivation, as the occluded eye fails to signal events carried by the other senses. A similar effect would be achieved by distorting the monocular image (*38*), applying a monocular inverting prism (*9*), or monocular delay (*10*), all of which introduce a sensory conflict and mimic the effects of monocular deprivation, despite leaving the strength of the visual stimulation unaltered. Repeated incongruence reduces the integration weights assigned to multisensory signals, thereby allowing for sensory recalibration (*39*). On this view, ocular dominance plasticity in adults may be reinterpreted as a recalibration response, triggered by the reduced top-down modulatory inputs conveyed by the pulvinar to V1. This perspective is in line with suggestions that the pulvinar plays a key role in cortical plasticity and the reshaping of functional organization in the visual cortex (*40*).

To conclude, our results stimulate a previously unexplored understanding of short-term monocular deprivation effects, which goes beyond local gain changes of evoked V1 responses, pointing to a functional reorganization of the visual processing network. We speculate that experience-dependent changes in early visual cortex depend on reducing the multimodal influences conveyed by the pulvinar, implying that the pulvinar may be the gatekeeper of adult sensory plasticity.

## MATERIALS AND METHODS

### Experimental design

Experimental procedures are in line with the principles of the declaration of Helsinki and were approved by the regional ethics committee [Comitato Etico Pediatrico Regionale—Azienda Ospedaliero-Universitaria Meyer—Firenze (FI)] and by the Italian Ministry of Health, under the protocol 0001650-15/01/2016-DGDMF-COD_UO-P “Plasticità e multimodalità delle prime aree visive: studio in risonanza magnetica a campo ultra alto (7T)” version no. 1 dated 11 November 2015. Written informed consent was obtained from each participant, which included consent to process and preserve the data and publish them in anonymous form. We analyzed data from two fMRI sessions, acquired before and after 2 hours of monocular deprivation. Twenty-five healthy volunteers with normal or corrected-to-normal visual acuity were tested. Sample size was set on the basis of the minimum number of participants (*N* = 18) required to reliably detect a medium-sized effect of monocular deprivation on fMRI signals (*6*, *7*). Because of image distortions, data from three and six participants were discarded from the first (repetition time; TR = 3 s) and second (TR = 1 s) fMRI dataset. This left respectively 22 participants for the TR = 3-s fMRI dataset (12 females mean age ± SEM = 26.5 ± 0.82, also included in the analyses of the DWI dataset) and 19 participants for the TR = 1-s fMRI dataset (12 females, mean age ± SEM = 26.5 ± 0.84).

### Short-term monocular deprivation

Monocular deprivation was achieved by patching the dominant eye for 2 hours. Similar in previous studies (*5*, *6*), we used a translucent eye-patch made of plastic material, allowing light to reach the retina (luminance attenuation: 0.07 logUnits). During the monocular deprivation, participants were free to walk, read, and use a computer, but they did not eat nor sleep.

### Binocular rivalry

Ocular dominance changes (before versus after deprivation) were evaluated with two brief binocular rivalry sessions, each comprising two 3-min runs, immediately before each fMRI session. The visual stimuli were presented on a 15-inch liquid crystal display (LCD) monitor viewed from 57 cm through anaglyph goggles (blue filter on the right lens and red filter on the left). The stimuli consisted of two oblique gratings (red and blue), oriented at ±45°, measuring 3° in diameter, with a spatial frequency of 2 cycles per degree (cpd) and 50% contrast. Equiluminance between the red and the blue gratings was achieved by reducing the intensity of the red channel based on photometric measurements (mean luminance of both the red and blue grating: 1.8 cd/m^2^). The dichoptic gratings were surrounded by a binocular sharp-edged white frame (encouraging fusion) and displayed against a uniform black background. During the experiment, participants continuously reported their perception by keeping one of three keys pressed: right or left arrows to report dominance of the clockwise or counter-clockwise oblique gratings and down-arrow key to report mixed percepts. Dominance phase durations shorter than 0.3 s (finger errors) and longer than 30 s (failed rivalry) were discarded. The dominant eye was defined as the eye showing the longer mean phase duration during a baseline binocular rivalry measurement performed in a separate training session.

We defined ocular dominance as the total time during which the stimulus presented in the dominant eye was perceived, divided by the total time during which the stimulus in either eye was perceived (which amounts to the total testing time minus the time during which a mixture of the two stimuli was perceived)

$$\text{OD}=\left(\frac{\text{TimeDE}}{\text{TimeDE}+\text{TimeNDE}}\right)*100$$ (1)

where OD stands for ocular dominance, DE for dominant eye, and NDE for nondominant eye. The effect of monocular deprivation was quantified as the OD difference before and after the 2-hour deprivation.

### MR system and sequences

Scanning was performed on a 7-T MRI system (SIGNA 7T-PISA, GE Healthcare, Milwaukee, WI, USA) equipped with a two-channel transmit and a 32-channel receiver head coil (Nova Medical, Wilmington, MA, USA).

Anatomical images were acquired at 0.8-mm isotropic resolution using a Magnetization Prepared RApid Gradient Echo sequence with the following parameters: echo time (TE) = 3.5 ms, GRE repetition time (TR_GRE_) = 7.3 ms, repetition time (TR_MPRAGE_) = 3380 ms, inversion time (TI) = 1100 ms, delay time (TD) = 1600 ms, flip angle  = 8°, receiving bandwidth (rBW) = 62.5 kHz, field of view (FOV) = 220 mm, matrix = 276 × 276, and slice thickness = 0.8 mm, 200 slices.

Two sets of fMR images were acquired at the same isotropic spatial resolution of 1.5 mm (matrix = 128 × 128, slice thickness = 1.4 mm, and slice spacing = 0.1 mm), rBW = 250 kHz, flip angle = 77°, ASSET = 2, and phase encoding direction anterior-to-posterior. The first fMRI dataset had TR = 3000 ms, TE = 23 ms, and number of volumes = 160. The second fMRI dataset had TR = 1000 ms, TE = 19.3 ms, and number of volumes = 300.

Each functional sequence included four initial dummy volumes to allow the stationarity of the signal. For each echo planar imaging (EPI)sequence, we acquired four additional volumes with the reversed phase encoding direction (posterior-to-anterior) used for distortion correction. We acquired each set of functional images (TR = 3 s first, followed by TR = 1 s) in resting-state with eyes closed.

For the study of structural connectivity, DWI datasets were acquired using a MUltiplexed Sensitivity-Encoding sequence with 2-mm isotropic resolution (FOV = 200 mm, matrix =100 × 100, and slice thickness = 2 mm). The other acquisition parameters were TR = 5500 ms, TE = 50.5 ms, 2 echoes, ASSET = 2, 32 noncollinear diffusion gradient directions with *b* value of 1000 s/mm^2^, and four volumes without *b* value = 0 s/mm^2^.

### MRI preprocessing

Preprocessing of anatomical and fMR images was performed with BrainVoyager (*41*) (version 20.6), FreeSurfer (*42*) (v6.0.0), FSL (*43*) (version 6.0.4), and ANTs (*44*). In addition, MATLAB (The MathWorks Inc.) was used to perform resting-state analyses.

Anatomical images were processed by a standard procedure for segmentation implemented in FreeSurfer [recon-all (*42*)]. Transformation matrices from individual participants’ T1 anatomical images to the Montreal Neurological Institute (MNI) template (*45*) were computed through an affine registration and a warping procedure using the antsRegistrationSyN.sh routine (*44*).

fMR images were corrected for slice time and motion (*41*) and undistorted using EPI images with reversed phase encoding direction, using the BrainVoyager COPE plug-in (*46*). We then exported the preprocessed images from BrainVoyager to NiFTi format. Each participants’ first functional acquisition (acquired right after the anatomical images) was aligned to the corresponding anatomy using a boundary-based registration algorithm (FreeSurfer bbergister function). All functional volumes were aligned to the same first functional acquisition with a rigid transformation (using antsRegistrationSyN.sh and antsApplyTransforms.sh) and then registered to the MNI space using the affine transformation and warp fields obtained from the anatomical registration (described above, matrices applied with antsApplyTransforms.sh).

fMR images with TR = 3 s were preprocessed as follows. After slice-time and motion correction, the fMRI time series were band-pass temporal filtered (0.01 to 0.1 Hz and minimum-order filter with a stopband attenuation of 60 dB); to minimize filtering artifacts, we excluded the first and last five TRs from further analysis. Next, we regressed out three main potential confounds (modeled by 14 regressors), as in previous work on pulvino-cortical interactions (*13*): (i) the six motion-parameter estimates and their temporal derivatives, (ii) the signal from the three ventricular spaces [average signal in the first, second, and third ventricles, defined by the recon-all routine (*42*)]. and (iii) the signal from the cerebral white matter [defined by the Harvard-Oxford subcortical atlas (*47*)].

fMR images with TR = 1 s were preprocessed in a similar way. After slice-time and motion correction, the fMRI time series were high-pass temporal filtered (0.0078 Hz and minimum-order filter with a stopband attenuation of 60 dB); to minimize filtering artifacts, we excluded the first and last five TRs from further analysis. Next, we regressed out three main potential confounds (modeled by 20 regressors): (i) the physiological parameters [respiration and cardiac pulsation preprocessed with the PhysIO Toolbox (*48*)], (ii) the signal from the three ventricular spaces [average signal in the first, second, and third ventricles, defined by the recon-all routine (*42*)], and (iii) the signal from the cerebral white matter [defined by the Harvard-Oxford subcortical atlas (*47*)].

DWI were preprocessed with QSIPrep (*49*). Preprocessing involved distortion correction, denoising using marchenko-pastur principal component analysis (MP-PCA), motion correction, and registration to anatomical template. Tensor fitting and reconstruction of the whole brain tractogram were performed with pyAFQ 1.3.6. The tractogram was generated with n_seeds = 8 using a constrained spherical deconvolution (CSD) model, and sampling was done from ROI masks. Optic radiations and pulvinar-V1 radiations were segmented on the basis of LGN, pulvinar, and V1 masks imported in pyAFQ. After successfully labeling tracts belonging to the optic radiation, outlier tracts were removed as a part of pyAFQ cleaning pipeline. Fractional anisotropy (FA) profiles for clean bundles were generated after resampling the tract length at 100 points. For each participant, we estimated the FA profiles before and after monocular deprivation and compared them with paired *t* tests. The associated two-tailed *P* values were evaluated against a false discovery rate (FDR) (*50*)–corrected 0.05 threshold.

### Statistical analysis

#### Functional connectivity analysis

Functional connectivity was estimated with both a within-participant and an aggregated subject approach. For the within-participant approach, we computed the Pearson’s correlation coefficient between the fMRI time series in pairs of ROIs (average time series of all voxels within the ROI). The correlation coefficients were Fisher transformed to ensure normality; the resulting values were compared before versus after monocular deprivation using paired *t* tests (two-sided *P* values). To check for possible changes in signal strength across time points, we also computed the Fourier spectrum of the fMRI time series in each ROI (in the 0.01- to 0.08-Hz frequency band) and compared its root mean square before and after deprivation using paired *t* tests (two-sided *P* values). With the aggregate-subject approach, we generated functional connectivity maps over the whole cortical surface. For this, we concatenated the *z*-scored individual participant’s fMRI time series and computed the Pearson’s correlation between the average time series in each ROI and the time series of each cortical voxels. We also computed voxel-wise differences of Pearson’s correlation coefficients before and after deprivation. We mapped the three coefficients (correlations before and after deprivation and correlation differences across deprivation) on a three-dimensional (3D) rendering of the cortical surface (fsaverage) and applied a spatial smoothing [full width at half maximum (FWHM) = 3 mm]. The two hemispheres were then registered to the same symmetric surface (fsaverage_sym), and the two correlation maps were averaged. The individual vertex correlation coefficients were associated with two-sided *P* values via Studentization (for the pre- and postdeprivation values) or using Fisher transformation (for the post-pre differences). The resulting surface maps (shown in Fig. 1 and figs. S1 and S2) were thresholded after FDR (*50*) correction of the two-sided *P* values (FDR-corrected thresholds for the pulvinar ROI: *p*_pre_ = 2.90 × 10^−3^; *p*_post_ = 2.80 × 10^−3^; *p*_difference_ = 1.45 × 10^−4^; LGN: *p*_pre_ = 1.80 × 10^−3^; *p*_post_ = 1.40 × 10^−3^; *p*_difference_ = NaN, meaning that no voxel passed the threshold; V1: *p*_pre_ = 3.00 × 10^−3^; *p*_post_ = 2.6 × 10^−3^; *p*_difference_ = 3.68 × 10^−5^) and cluster corrected (cluster size = 50 vertices). We accompanied these maps with outlines of the main clusters displaying robust connectivity at baseline (|*r*| > 0.2 and cluster = 500 vertices). We also visualized volume maps (Fig. 2), where correlation values before and after deprivation were thresholded after FDR (*50*) correction of the *P* values (thresholds: *p*_pre_ = 9.87 × 10^−6^, *p*_post_ = 9.87 × 10^−6^; no cluster correction).

#### Effective connectivity analysis

From the high-TR fMR images (TR = 1 s, average time series of all voxels within the pulvinar, LGN, and V1 ROIs), we estimated effective connectivity with a within-subject approach using DCM (*26*) in the rDCM toolbox implementation (*51*, *52*) [available as part of the TAPAS software collection (*53*)]. The rDCM routine returned a value of “negative free energy,” indicative of the trade-off between accuracy and complexity of the model. We compared these values across two models: one representing the fully connected network, i.e., bidirectional connections between pulvinar and V1, LGN, and V1 and between pulvinar and LGN; and one without the connections between pulvinar and LGN (which are not supported by anatomical evidence). Performance of the latter was systematically better across all participants; we therefore reported its connectivity estimates (the “A matrix”) in the main text (Fig. 2); fig. S4A also reports the (very similar) connectivity estimates for the fully connected model. The significance of the individual connections was assessed with one-sample *t* tests across participants, and the associated two-tailed *P* values were evaluated against an FDR (*50*)–corrected 0.05 threshold.

We further tested the effect of monocular deprivation on effective connectivity with a two-by-two ANOVA for repeated measures, with main factors time (before and after deprivation) and directionality (e.g., pulvinar-to-V1 and V1-to-pulvinar). This was followed up with post hoc *t* tests, and the associated two-tailed *P* values were evaluated against an FDR (*50*)–corrected 0.05 threshold.

We complemented this DCM analysis with a simpler cross-correlation of the same fMRI time series. This was implemented with the standard xcorr MATLAB function set to the “normalize” option, which returned cross-correlation coefficients normalized by the autocorrelations. To test whether the asymmetry of the curves changed after deprivation, we took the average cross-correlation at the three negative and positive lags closest to zero ([–3 –2 –1] and [+1 +2 +3]) and evaluated their difference before versus after deprivation with a series of paired *t* tests across participants. The associated two-tailed *P* values were evaluated against an FDR (*50*)–corrected 0.05 threshold.

### ROI definition

All ROIs were defined in the MNI space, to which our functional scans were registered. For each subject and fMRI acquisition, we extracted the average BOLD signal from all voxels within the ROI (using the fslmeants function).

The pulvinar ROI was taken from a published atlas (*21*) defined from DWI. The LGN was taken from the Natural Scenes Dataset, which outlined it based on a combination of functional data (retinotopic mapping experiments) constrained with anatomical features (*25*). The V1 ROI was taken from the Glasser cortical parcellation atlas (*24*), defined in the fsaverage surface space, and projected to the MNI volume (https://identifiers.org/neurovault.collection:1549).

### Pulvinar sub-ROI definition with visually evoked activations

The same participants took part in an additional experiment to identify visually responsive regions within the thalamus. Visual images were presented with MR-compatible goggle set (VisuaStimDigital, Resonance Technologies, Los Angeles, USA), connected to a computer placed in the control room. The goggles covered a visual field of ~32 × 24°, with a resolution of 800 × 600 pixels, mean luminance of 25 cd/m^2^, and frame rate of 60 Hz. We used similar stimuli as in (*7*): Band-pass noise stimuli (peak 0.4 cpd and bandwidth 1.25 octave), refreshing at 8 Hz, presented in a block design with 9-s stimulation, followed by 12 s of blank screen (except for a 0.5° red fixation spot that was always visible). The contrast of the stimuli was variable (6, 12, 25, 50, and 100%); each run included 10 blocks with the five contrast levels presented in ascending order and repeated twice.

The experiment consisted of a total of four runs with 70 TRs: two runs in passive viewing followed by two runs with attention allocated to the left or right half of the visual field (one side per run) to detect small color changes (blue blobs with 1° radius, presented for four monitor frames, i.e., 67 ms) superimposed to the band-pass noise stimulus and occurring at random times and locations during the 9 s of stimulus presentation. Participants counted the number of transients occurring in each block on the cued side, which varied randomly between 2 and 6, and they reported by keypress whether it was smaller or larger than 4.

fMRI time courses were slice-time and motion corrected. For each voxel within the pulvinar ROI, we computed the BOLD signal variations (in % signal change) with each stimulus presentation, forming epochs of 7 TRs (21 s) referenced to the first TR. The mean over time in each epoch defined the event-related response. We averaged these event-related responses across the 20 epochs (10 per run) and our 22 participants to evaluate the strength of the BOLD modulation in each voxel of the pulvinar region.

These maps were used to identify maximally responsive voxels in each condition: passive viewing versus with attention allocated to the stimulus area. We thresholded maps at 0.25% BOLD signal change and defined binary masks of activated voxels after applying a small spatial smoothing (1 voxel FWHM). Voxels responding in passive viewing or both conditions were assigned to a first region, which was ventrally located; voxels selectively responding to attended stimuli were assigned to a second region, which was localized in a more dorsal territory. This produced two nonoverlapping masks of 1036 and 1111 voxels (relatively dorsal and ventral regions, respectively, with the number representing voxels in both hemispheres), collectively covering 55.4% of the anatomically defined pulvinar ROI (*21*) (3876 voxels across both hemispheres).
